# Paradoxical augmented relapse in alcohol-dependent rats during deep-brain stimulation in the nucleus accumbens

**DOI:** 10.1038/tp.2016.100

**Published:** 2016-06-21

**Authors:** R Hadar, V Vengeliene, E Barroeta Hlusicke, S Canals, H R Noori, F Wieske, J Rummel, D Harnack, A Heinz, R Spanagel, C Winter

**Affiliations:** 1 Department of Psychiatry and Psychotherapy, University Hospital Medical Faculty Carl Gustav Carus, Technische Universität Dresden, Dresden, Germany; 2 Institute of Psychopharmacology, Central Institute of Mental Health, Medical Faculty Mannheim, Heidelberg University, Mannheim, Germany; 3 Cellular and Systems Neurobiology Unit, Instituto de Neurociencias, Consejo Superior de Investigaciones Científicas and Universidad Miguel Hernández, Sant Joan d'Alacant, Spain; 4 Department of Neurology, Charité Universitätsmedizin Berlin, Berlin, Germany; 5 Department of Psychiatry and Psychotherapy, Charité Universitätsmedizin Berlin, Berlin, Germany

## Abstract

Case reports indicate that deep-brain stimulation in the nucleus accumbens may be beneficial to alcohol-dependent patients. The lack of clinical trials and our limited knowledge of deep-brain stimulation call for translational experiments to validate these reports. To mimic the human situation, we used a chronic-continuous brain-stimulation paradigm targeting the nucleus accumbens and other brain sites in alcohol-dependent rats. To determine the network effects of deep-brain stimulation in alcohol-dependent rats, we combined electrical stimulation of the nucleus accumbens with functional magnetic resonance imaging (fMRI), and studied neurotransmitter levels in nucleus accumbens-stimulated versus sham-stimulated rats. Surprisingly, we report here that electrical stimulation of the nucleus accumbens led to augmented relapse behavior in alcohol-dependent rats. Our associated fMRI data revealed some activated areas, including the medial prefrontal cortex and caudate putamen. However, when we applied stimulation to these areas, relapse behavior was not affected, confirming that the nucleus accumbens is critical for generating this paradoxical effect. Neurochemical analysis of the major activated brain sites of the network revealed that the effect of stimulation may depend on accumbal dopamine levels. This was supported by the finding that brain-stimulation-treated rats exhibited augmented alcohol-induced dopamine release compared with sham-stimulated animals. Our data suggest that deep-brain stimulation in the nucleus accumbens enhances alcohol-liking probably via augmented dopamine release and can thereby promote relapse.

## Introduction

The adverse effects of alcohol consumption cause enormous health, social and economic burdens in societies worldwide. In its fifth edition, the Diagnostic and Statistical Manual of Mental Disorders (DSM-5) integrated alcohol abuse and alcohol dependence into one single disorder referred to as alcohol-use disorder.^1^ The behavioral characteristics of this disorder are compulsive alcohol use, craving and relapses that can be observed even years following abstinence. Given that alcoholism is the result of cumulative responses to alcohol exposure, the genetic make-up of individual and environmental perturbations over time,^2^ patient populations can differ largely in their treatment responses and some patients are resistant to all available treatment options.

The mechanisms of action of currently approved pharmacotherapies include interfering with alcohol's metabolism, leading to an aversive physiological reaction following alcohol intake (disulfiram), targeting the reward pathways in the brain in an attempt to reduce alcohol reward and craving for alcohol (naltrexone and nalmefene) and interfering with a hyperglutamatergic system in order to avoid relapses (acamprosate).^3, 4^ However, the overall effect size of these medications is moderate and high relapse rates are still observed, outlining the need for the development of new therapeutic strategies.

In recent years, deep-brain stimulation (DBS) has been proposed as an alternative therapy for various neuropsychiatric conditions, including severe and chronic alcohol dependence.^5, 6, 7^ In an initial report, a remission of alcohol addiction following high-frequency stimulation in the nucleus accumbens (NAc) was observed in a patient treated for severe anxiety disorder and secondary depressive disorder.^8^ A few case reports in humans published since this initial observation complement and support the notion that high-frequency DBS in the NAc may be a new treatment option for very severe cases.^5, 6, 9, 10^ Furthermore, a small number of animal studies suggest that high-frequency DBS in the NAc can reduce alcohol intake and preference in rats.^11, 12, 13^ However, these preclinical studies provide limited conclusions, as acute stimulation protocols were applied to rats that were not alcohol-dependent.

Although the exact mechanism remains obscure, DBS appears to act not only locally, but also at a systems level, modulating neural network activity associated with the stimulation target via the activation of afferent and efferent axons, and may thereby recalibrate pathological alterations in network function. As such, DBS is more than a therapeutic technique, as it also holds promise for promoting our understanding of the underlying neurobiological substrates of aberrant behaviors.^14, 15^

Although clinical trials on DBS in alcohol-dependent patients have been initiated, it is becoming increasingly clear that patient recruitment and ethical considerations (for example, sham control stimulation) are a major challenge and that we have very limited knowledge about the target region and stimulation parameters. In this difficult situation, translational investigations that use animal models of alcohol addiction to study the mechanisms by which DBS exerts its effects and identify and characterize alternative stimulation targets and parameters are warranted.^16^

Here we sought to study the effects of chronic bilateral stimulation of the NAc on relapse-like drinking in a rat model. For this purpose, rats were subjected to long-term voluntary alcohol consumption in a four-bottle procedure, repeatedly interrupted with abstinence periods. In rats that had long-term voluntary access to alcohol followed by deprivation for several weeks, the re-presentation of alcohol leads to relapse-like drinking—a temporal increase in alcohol intake over baseline drinking. This robust phenomenon is called the alcohol-deprivation effect (ADE).^17, 18, 19^ Our previous research has demonstrated that rats that undergo long-term alcohol drinking for several months with repeated deprivation phases develop a compulsive drinking behavior during a relapse situation, characterized by insensitivity to taste adulteration with quinine, a loss of circadian drinking patterns during relapse-like drinking, and a shift toward drinking highly concentrated alcohol solutions to rapidly increase blood alcohol concentrations and achieve intoxication. In addition, alcohol-dependent rats that derive from this model show tolerance and physical as well anxiety-related withdrawal symptoms. In summary, alcohol-dependent rats derived from this model show symptoms used in DSM-IV and DSM-5 as diagnostic criteria.^19, 20, 21, 22, 23^ This animal model has been used in numerous preclinical and translational alcohol studies and has helped identify new treatment targets with good predictive validity.^2, 22–25^

Our DBS experiments were translationally designed to best match the human condition; that is, we used the same stimulators as usually applied in humans and performed chronic-continuous stimulation to the NAc before and during a relapse situation. To our surprise, we consistently observed a paradoxical effect. Thus, animals had enhanced alcohol consumption and exhibited more pronounced relapse behavior than sham-stimulated control rats. In order to validate and understand this paradoxical effect, we replicated previous animal experiments that showed a reduction in the ADE in rats that were not alcohol-dependent.^12^ Using then a series of experiments that combined DBS (electrical stimulation) with functional magnetic resonance imaging (es-fMRI)^25, 26, 27^ and neurochemical measurements^28,29^, we were able to offer a possible explanation of the paradoxical DBS effect pertaining to dopamine (DA) release properties within the NAc.

## Materials and methods

### Animals

Male Wistar rats from our breeding colony at the Central Institute of Mental Health in Mannheim were used in all experiments. Animals were single-housed in standard hanging cages at 21±1 °C and 50±5% relative humidity on a 12-h light/dark cycle, with lights on at 0700 hours. Animals were provided with standard rodent food and tap water *ad libitum*. Experiments were conducted in accordance with the European Union guidelines on the care and use of laboratory animals and after approval of the local ethic committees (Regierungspräsidium Karlsruhe and Dresden). We follow the 3-Rs principle, and all efforts were made to minimize animal suffering and to reduce the number of animals used.

### Long-term alcohol consumption with repeated deprivation phases

After 2 weeks of habituation to the animal room, rats were given *ad libitum* access to tap water and 5, 10 and 20% ethanol solutions (v/v) in their home cages. The first 2-week deprivation period was introduced after 8 weeks of continuous ethanol availability. After this deprivation period, rats were given access to ethanol again. This access was further interrupted repeatedly with deprivation periods in a random manner (that is, the duration of following drinking and deprivation phases was irregular: ~4–6 and 2–3 weeks, respectively). The long-term voluntary ethanol-drinking procedure including all deprivation phases lasted a total of ~1 year.

Our earlier studies have demonstrated that voluntary alcohol drinking for 2 months does not lead to the occurrence of addictive-like behavior ('alcohol-non-dependent rats'). However, following long-term alcohol consumption with repeated deprivation periods, the ADE is characterized by enhanced alcohol-'liking‘ and incentive salience for the drug ('wanting'), the loss of predictability of the drinking behavior and loss of a typical circadian drinking pattern;^19, 20, 21^ thus, rats that derive from this procedure are labeled as 'alcohol-dependent'. Hence, all experiments with alcohol-non-dependent rats started at the end of the first alcohol-deprivation period, whereas rats subjected to the long-term drinking procedure with repeated deprivation periods for ~1 year were used as alcohol-dependent rats.

### Drinkometer system

The drinkometer system has been developed together with TSE Systems (Bad Homburg, Germany). It enables continuous long-term monitoring of liquid consumption by amount and time in a standard rat home cage (Eurostandard Type III). The system is equipped with four drinking stations to allow liquid choice. The drinking station consists of a glass vessel containing the liquid and a high-precision sensor for weighing the amount of liquid removed from the glass vessel. Spillage and evaporation are minimized by using special bottle caps. Monitoring of all drinking stations is carried out with a computer. The system features ultrahigh resolution—down to 0.01 g. The whole system is mounted to a custom-made free-swinging steel frame in order to avoid any environmental disturbances. The drinkometer system measures the weight of a vessel in 200 ms steps and saves it in 1-s steps. The normal sampling can be set with minimum 1-min intervals. For the present study, sampling was performed at 5-min intervals.

### DBS experiments

For DBS experiments, monopolar platinum iridium electrodes (250 μm in diameter, PlasticsOne, Roanoke, VA, USA) were used. The stereotaxic surgery was conducted under isoflurane anesthesia (CP Pharma, Braunschweig, Germany; 1.9–2.2%). Stimulating electrodes were implanted bilaterally into the NAc shell (NAcs), caudate putamen (CPu) and infralimbic (IL) cortex. We aimed for the NAcs as the clinicians involved in the previously published human DBS studies ensured us that their stimulation electrodes were located in the NAcs (personal communications with Volker Sturm and Hans-Joachim Heinze and studies by Heinze *et al.*,^5^ Heldmann *et al.*^9^ and Voges *et al.*^10^). For the NAcs, the following coordinates with respect to the bregma were used: 1.6 mm anterior to bregma, ±0.8 mm lateral to the midline and 7.6 mm ventral to the dura (Paxinos and Watson, Atlas). These coordinates define the desired target region for stimulation. For DBS in other brain sites, the coordinates are given in Supplementary Information. Following the stereotactic procedure, a transection of the abdominal wall was made for the placement of the Medtronic Interstim II neurostimulator device (Model 3058). Lead wires were retrogradly tunneled and connected to the stimulation device. For sham-stimulated control rats, a dummy equivalent to the neurostimulator device in size, form and weight was implanted. DBS was then applied at the constant voltage mode, 130 Hz and 90 μs pulse duration, 5.25 V.

#### Chronic-continuous DBS

In order to study the effect of chronic-continuous DBS in the NAcs on the expression of the ADE in alcohol-dependent rats, they were divided into four groups (*n*=10–15, see the figures for exact number of animals used in each group). Half of the animals were tested in the drinkometer device, whereas the other half remained in their home cages. Animals were assigned into groups in such a way that the mean baseline total ethanol intake between DBS and sham-stimulated groups, as well as the intake of every solution separately (that is, water, 5% ethanol, 10% ethanol and 20% ethanol), was matched. Baseline drinking was monitored for 6 days. After the last day of baseline measurement, the ethanol bottles were removed from the cages, leaving the animals with free access to food and water for ~3 weeks. On the second week of deprivation, two groups of rats were bilaterally implanted with sham electrodes and the remaining groups were implanted with stimulation electrodes in the NAcs. After the surgery, rats were allowed to recover for at least 1 week. Thereafter, animals were subjected to chronic-continuous DBS. Stimulation started 3 days before alcohol re-presentation and continued for 4 post-abstinence days. Alcohol and water consumption was monitored throughout the stimulation period and two more days post stimulation. DBS in the CPu and IL cortex is described in Supplementary Information.

### Acute DBS

In order to compare the effect of acute DBS on the post-abstinence drinking between non-dependent and dependent animals, a separate batch of rats was divided into groups (*n*=6–9, see the figures for exact number of animals used in each group) in such a way that the mean baseline total ethanol intake between groups was similar. On the second week of alcohol deprivation, dummy or stimulation electrodes were implanted bilaterally into the NAcs, and animals were allowed to recover for at least 1 week. Thereafter, animals from one group were subjected to acute stimulation protocol, whereas the other group served as the control sham DBS group. Stimulation started 1 h before alcohol re-presentation and lasted for 24 post-abstinence hours.^12^ Intake of alcohol and water was measured during this first post-abstinence day.

### Biophysical modeling

For modeling the patterns of electric field potentials within NAcs during DBS, the two-dimensional coronal (anterior to bregma=1.2) and sagittal (lateral to the midline=1.2) geometry of the NAc was digitalized. On the basis of the experimental stimulation parameters, an elliptic Poisson partial differential equation describing the electric potential fields in conductive media –div(σ.∇ (V))=*q* (with *E*=−∇ (*V*)) and a hyperbolic system of partial differential equations describing the propagation of electromagnetic waves within non-homogeneous anisotropic media ∇ × (*μ_r_*^−1^.(∇ × *E*))+1/*c*^2^ *ε_r_* (∂^2^ *E*)/(∂*t*^2^)=−*μ*_0_ ∂σ*E*/∂*t* were parameterized and numerically simulated under Dirichlet boundary conditions (hV=*r*; *h*=1, *r*=5.25 V for boundaries at the electrode and *h*=1, *r*=0 else) by finite element method in MATLAB. *V* denotes the electric potential, *E* the electric field, *σ* the conductivity of the media, *q* the current source and *μ_0_, μ_r_, ε_r_* and *c* are the classical parameters of Maxwell equations for permeability in vacuum, permeability in the considered medium, the relative permittivity and the speed of light. The electromagnetic tissue parameters were obtained from the Andreuccetti online database. In particular, the conductivity, permittivity and permeability of the NAc were approximately obtained from the gray matter value provided for 130 Hz as *σ*=9.15 × 10^−2^ S/m and *ε_r_*=2.46 × 10^6^ F/m and *μ_r_*=9.3 × 10^−10^ H/m, respectively.

### Es-fMRI

Glass-coated carbon fiber (7 μm diameter; Goodfellow Cambridge, Huntingdon, UK) bipolar electrodes were used for brain stimulation in fMRI experiments to avoid image artifacts^27^ and to preserve the quality of MR images by allowing functional activations in the vicinity of the implanted electrode.^28, 29^ The electrodes consisted of a bundle of fibers inserted into a theta-shaped glass capillary (World Precision Instruments, Sarasota, FL, USA) previously pulled to form 10-mm-long pipettes with ∼200-μm tip diameter and adjusted to produce an electrical impedance of ∼40 kΩ. A regular wire with a pin connector was attached to the pipette, connected to the carbon fibers using silver conductive epoxy resin (RS Components, London, UK), and isolated with clear epoxy resin. Afterward, the tip was bent in a flame to form a 90º angle to minimize implant size, and thereby allow close proximity between the MRI array coil and the head of the animal.

For es-fMRI measurements, a separate batch of alcohol-dependent rats, deprived from alcohol for 3 weeks, were used (*n*=6). Electrodes were implanted into the NAcs as described above. Thereafter, rats were placed in a custom-made animal holder with adjustable bite and ear bars, positioned on the magnet bed and constantly supplied with 0.8 l min^−1^ O_2_ with a face mask. Temperature was kept between 37.0 and 37.5 °C through a water heat pad. Body temperature, heart rate, SpO_2_ and the breathing rate were monitored throughout the session (MouseOx, Starr Life Sciences, Oakmont, PA, USA). The experiments were carried out in a horizontal 7 Tesla scanner with a 30-cm-diameter bore (Biospec 70/30, Bruker Medical, Ettlingen, Germany).

Acquisition was performed in 15 coronal slices using a Gradient Echo - Echo Planar imaging (GE-EPI) sequence applying the following parameters: field of view (FOV), 25 × 25 mm; slice thickness, 1 mm; matrix, 96 × 96; segments, 1; flip angle (FA), 60° echo time (TE), 15 ms; repetition time (TR), 2000 ms. T2-weighted anatomical images were collected using a rapid acquisition relaxation enhanced sequence (RARE): FOV, 25 × 25 mm; 15 slices; slice thickness, 1 mm; matrix, 192 × 192; TEeff, 56 ms; TR, 2 s; RARE factor, 8. A 1H rat brain receive-only phase array coil with integrated combiner and preamplifier, and no tune/no match, was employed in combination with the actively detuned transmit-only resonator (Bruker BioSpin MRI, Rheinstetten, Germany). For stimulation during fMRI recordings, charge balanced biphasic 0.1 -ms duration pulses of 150 μA with 90 μs pulse duration were delivered at 130 Hz with a constant current source and a pulse generator (STG2004, Multi Channel Systems, Reutlingen, Germany). The protocol consisted of 10 repetitions of stimulation ON periods lasting for 8 s, followed by OFF periods of 26 s (300  s per protocol); protocols were repeated three times.

### Neurochemical analysis

Tissue high-performance liquid chromatography (HPLC) analysis (for details see Hadar *et al.*^28^). Alcohol-non-dependent and -dependent rats, which underwent an acute stimulation protocol, were used for tissue HPLC analysis. After completion of the stimulation experiments, rats were decapitated and their brains were stored at −80 °C. Micropunches of NAcs, CPu and mPFC were taken from 500-μm-thick coronal sections. Tissue was homogenized and centrifuged at 17 000 *g* at 4 °C for 10 min. Aliquots of the supernatants were used for analyses of amino acids and monoamines. Monoamines were separated by HPLC and electrochemically detected (Chromsystems Instruments & Chemicals, Munich, Germany) at an electrode potential of 0.8 V. Glutamate and GABA were pre-column-derivatized with o-phthalaldehyde-2-mercaptoethanol and then separated on a HPLC column. Derivatized amino acids were detected by their fluorescence at 450 nm after excitation at 330 nm.

*In vivo* microdialysis (for details see Winter *et al.*^29^). Rats from a separate batch of alcohol-dependent rats were divided into two groups (*n*=6–7) after 3 weeks of abstinence in such a way that the mean baseline total ethanol intake between groups was similar. For the microdialysis experiment, combined electrode-guide cannulas were unilaterally implanted into the NAcs so that the tip of the guide cannula ended 2.0 mm above while the tip of the electrode was placed within the target region. The assembly was anchored with two stainless-steel screws and dental acrylic. Dialysis probes (CMA11, Axel Semrau, Sprockhövel, Germany) with 2-mm active membrane were introduced into the guide cannula 1 h before the beginning of the dialysis experiments. Electrodes were connected via an isolated cable system to an isolated stimulator (Multi Channel Systems). Probes were perfused with artificial cerebrospinal fluid with a constant flow rate of 1.1 μl per min. Samples were collected at 20 min intervals in 5 μl of 1 M perchloric acid for immediate analysis of extracellular concentrations of DA via HPLC and electrochemical detection (LC-4C, BAS, West Lafayette, IN, USA). Before brain stimulation, samples were collected until at least four consecutive, stable values were measured. Thereafter, in one group of rats NAcs DBS was switched on, whereas control animals received no stimulation. After 1 h of DBS/sham DBS, all rats were given an additional three bolus injections of ethanol (10%v/v) in ascending doses (0.5, 1.0, 1.5 mg kg^−1^ body weight) in 40-min intervals.

### Data analysis

Data obtained from the chronic-continuous and acute stimulation experiments on total daily ethanol and water intake was analysed using a two-way analysis of variance with repeated measures (factors were as follows: stimulation (sham versus DBS) and time (baseline versus ADE days)). Whenever significant differences were found, *post hoc* Student Newman–Keuls test was performed.

To investigate the effects of chronic-continuous DBS on the drinking patterns measured in the drinkometer device, we used an analogous approach to our previous studies.^20, 21, 30^ We utilized Fourier analysis to characterize the recurrent drinking events within the drinkometer data sets. The Fourier analysis provides a function 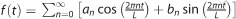 with the maximum likelihood to describe the amount of water drinking or drinking of ethanol mixtures in the Drinkometer system during the measurement interval *L*. On the basis of the frequencies 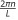 and the Fourier coefficients 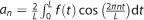 and 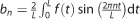, this approach provides approximate measures for drinking frequencies ('wanting'), drinking peak times and peak intake ('liking').^20, 21^

fMRI data were analyzed offline using self-developed software, which included Statistical Parametric Mapping package (SPM8, www.fil.ion.ucl.ac.uk/spm), Analysis of Functional NeuroImages (http://afni.nimh.nih.gov/afni) and FSL Software (FMRIB, http://fsl.fmrib.ox.ac.uk/fsl/). After linear de-trending, temporal (0.015–0.2 Hz) and spatial filtering (3 × 3 gaussian kernel of 1.5 sigma) of voxel time series, a general linear model was applied with a simple boxcar model shifted forward in time, typically by 2 s or a boxcar convolved with a gamma probability density function (hemodynamic response function). Functional maps were generated from voxels that had a significant (*P<*0.001) component for the model and they were clustered together in space (cluster size=14; calculated with Monte Carlo simulation implemented in Analysis of Functional NeuroImages). Region of interests extracted using a rat atlas registered to the functional images were used to compute the relative volume of brain tissue activated (number of active voxels divided by the total number of voxels in the region). Group maps showing the probability for each voxel of being activated by a particular stimulation protocol were also calculated. A voxel probability of 1 means that all animals used in the study showed coincident activation of that particular voxel to a certain protocol.

Data obtained from the tissue HPLC analysis was analyzed using a two-way analysis of variance (factors were as follows: stimulation (sham versus DBS) and stage of dependence (alcohol-non-dependent versus alcohol-dependent)). Data from the microdialysis experiment was analyzed by use of a two-way analysis of variance with repeated measures (factors were as follows: stimulation and time).

The chosen level of significance for all experiments was *P<*0.05.

## Results

### Chronic-continuous NAcs DBS leads to a paradoxical augmented relapse behavior in alcohol-dependent rats

**Figure 1:**
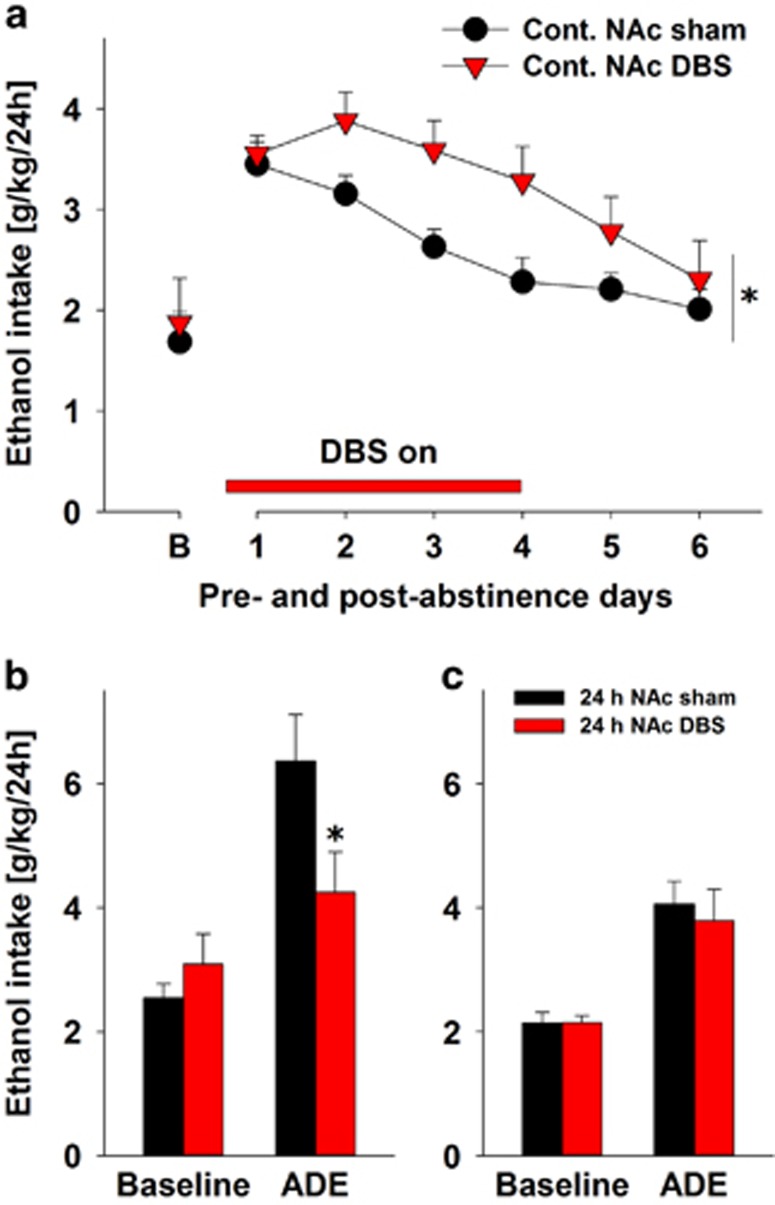
Paradoxical effect of DBS on alcohol relapse-like drinking (ADE). (**a**) Total ethanol intake (g kg^−1^ per day) before and after an alcohol-deprivation period of 3 weeks in alcohol-dependent rats in the sham-stimulated control group (*n*=15) and in the bilateral nucleus accumbens shell (NAcs) deep-brain stimulation (DBS) group (*n*=10). The last week measurement of ethanol intake is given as baseline drinking—'B'. Chronic-continuous bilateral NAcs stimulation (Cont. NAc DBS) started 3 days before the end of the abstinence phase and continued for four post-abstinence days (horizontal bar). (**b**) First day of ADE in alcohol-non-dependent sham-stimulated (*n*=9) and in 24-h bilateral NAc DBS (*n*=6) rats. (**c**) First day of ADE in alcohol-dependent sham-stimulated (*n*=8) and in 24-h bilateral NAc DBS (*n*=6) rats. Twenty-four-hour bilateral NAcs stimulation (24-h NAc DBS) started 1 h before the end of abstinence phase and continued for 24 post-abstinence hours. * indicates significant difference from the control sham group. Data are presented as means ±s.e.m., *P<*0.05.

**Figure 2:**
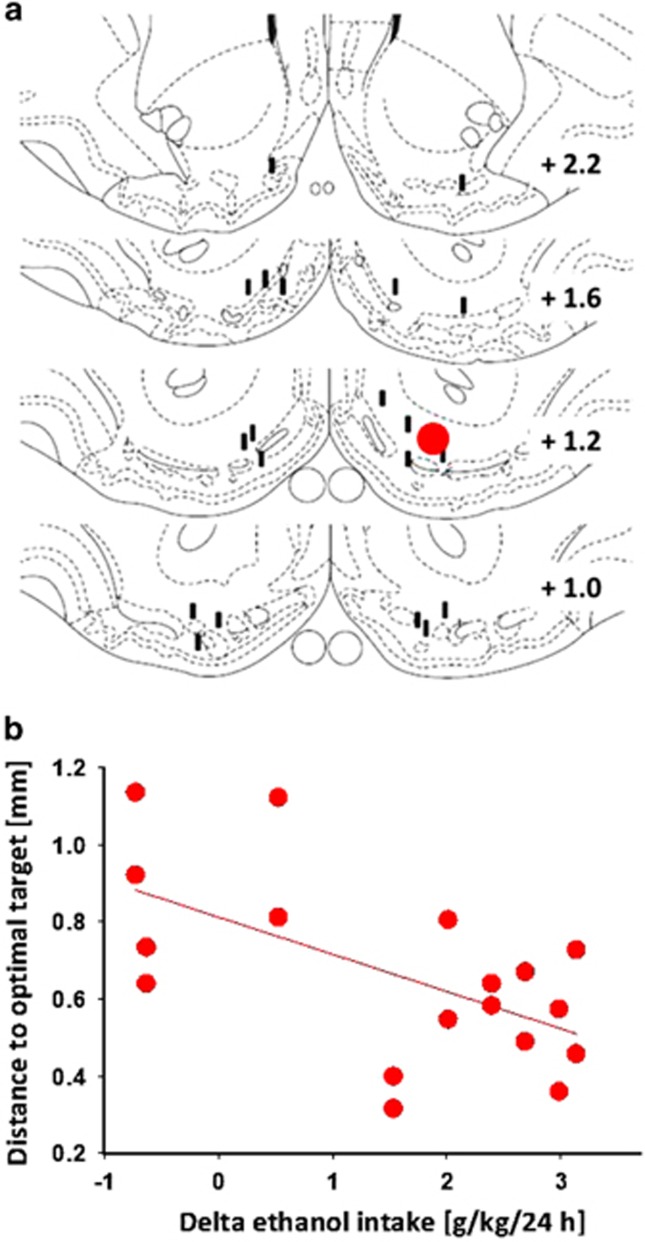
Localizations of the stimulation electrodes. (**a**) Coronal sections from the caudal to rostral regions of the nucleus accumbens (NAc) showing desired target region (red) and localizations of the tip of the stimulation electrodes (black) within the NAcs in alcohol-dependent continuously stimulated rats. (**b**) Negative correlation (Rho=0.60; *P<*0.01) between the distance of each electrode tip to the desired target within the NAcs and individual alcohol intake in alcohol-dependent continuously stimulated rats. Respective delta ethanol intake was calculated by subtracting average ethanol intake of the first four alcohol-deprivation effect (ADE) days from that of the last four baseline days.

Our initial working hypothesis was that chronic-continuous DBS within the NAcs would reduce relapse-like drinking in an animal model for alcohol addiction. For testing this hypothesis, we assessed ethanol intake before and after an alcohol-deprivation period in long-term alcohol-drinking control rats that were sham-stimulated and a DBS group of rats. Chronic-continuous stimulation started 3 days before the end of the abstinence phase and continued for four post-abstinence days. Two-way analysis of variance for repeated measures revealed a significant increase in alcohol intake after a deprivation phase in both DBS and sham DBS groups as compared with baseline drinking, indicating a robust ADE (factor time: F(6,138)=19.4, *P<*0.0001). However, DBS of NAcs significantly increased the expression of ADE when compared with sham-stimulated rats (factor stimulation: F(1,23)=4.5, *P<*0.05; Figure 1a). Verifying the localizations of the tip of the stimulation electrodes within the NAcs of the DBS group and calculating the distance of each electrode tip to the desired target within the NAcs, we found that the mean individual alcohol intake correlated negatively with the distance of the electrode tip placement to the desired target within the NAcs (Figure 2).

In the drinkometer device, high temporal resolution of drinking behavior is achieved that also allows the measurement of frequency of approaches to the alcohol bottles. We have previously demonstrated that a relapse in rodents is also characterized by an increased frequency of approaches to the alcohol bottles, which is interpreted as an increased alcohol 'wanting‘, whereas increased drinking during an approach to the bottle (amplitude) is seen as increased 'liking‘.^20, 21^ In the drinkometer device, a paradoxical effect with augmented expression of ADE was again measured following NAcs DBS (factor time × stimulation interaction: F(6,66)=2.5, *P<*0.05; data not shown). The drinkometer data were then submitted to subsequent Fourier analysis. A significant increase in the frequency of approaches to the high-concentrated alcohol solutions during the ADE was measured in control sham-stimulated animals. On the first day of the ADE the frequency of approaches was 14-fold higher over the baseline for 10% ethanol and 10-fold increased for 20% ethanol, whereas frequency of approaches to the 5% ethanol solution and water bottle did not change, indicating an increased 'wanting‘ during ADE, especially for higher-concentrated ethanol solutions (Table 1). Chronic-continuous DBS either tended or significantly reduced the frequency of approaches to the bottles of more concentrated ethanol solutions (10 and 20%), but increased the amplitude of a drinking occasion by fourfold for the highest-concentrated ethanol solution (Table 1). In summary, chronic-continuous DBS produced a robust augmentation of relapse-like drinking. This phenomenon was mainly driven by enhanced alcohol 'liking' at high alcohol concentrations.

The observed paradoxical effect is in stark contrast to previous reports that showed a reduction of alcohol intake and preference^11, 12^ as well as a reduction of the ADE.^12^ Therefore, we decided to replicate the study by Henderson *et al.*^12^ In this representative study, an acute 1-day stimulation protocol was used during the re-introduction of ethanol solutions after the first period of deprivation and a strong reduction of the ADE was observed. We were able to fully replicate this finding by showing that the first ADE day was significantly reduced by acute bilateral stimulation of the NAcs (factor time × stimulation interaction: F(1,13)=5.4, *P<*0.05)—during the ADE, stimulated rats consumed ~1/3 less alcohol than sham-stimulated rats (Figure 1b). However, when we applied this acute stimulation protocol to animals that had repeated deprivation periods and showed compulsive drinking behavior (that is, dependent rats), DBS did not produce a reduction on the ADE anymore (factor time × stimulation interaction: *P*=0.57; Figure 1c). It should be noted that ADE was not as pronounced in dependent rat groups as it was in non-dependent animals (Figures 1b and c). The reason for this could have been different ages of animals—younger animals (non-dependent) are known to tolerate stressful situations more easily than older animals (dependent). The acute sham as well as the DBS stimulation is a stressful procedure, and it is well known that stress may lead to reduced drinking. Nevertheless, post-abstinence drinking was significantly higher in both non-dependent and dependent rats NAcs (factor time: F(1,13)=18.9, *P<*0.001 and F(1,12)=57.7, *P<*0.001 for non-dependent and dependent rat groups, respectively).

What could be the biological reason for the observed paradoxical effect of DBS in alcohol-dependent rats? To answer this question we first analyzed the local as well as the network effects of DBS stimulation in alcohol-dependent rats.

### Electrical stimulation of the NAcs affects the stimulation site and a well-defined set of associated brain regions

**Figure 3:**
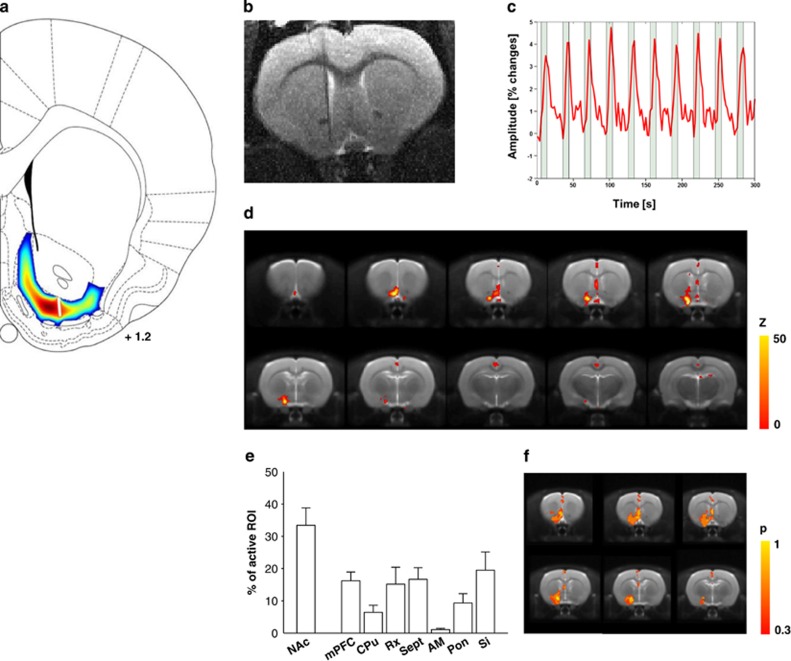
Electrical stimulation of the nucleus accumbens shell (NAcs) affects stimulation site and a well-defined set of associated brain regions. (**a**) Biophysical simulation of NAcs stimulation with the same stimulation parameters as used for the behavioral experiments. The propagation of the induced potential waves within the accumbal tissue as a dissipative and approximately isotropic media is decreasing with growing distance from the stimulation site and vanishes toward the boundaries of the simulation domain. Therefore, it is safe to assume that interactions with neighboring structures are negligible. The stimulation domain was defined by the surface spanned through the boundary of the NAcs as given by the Paxinos and Watson atlas of the rat brain. (**b**) A representative anatomical T2-weighted image with a location of a carbon-fiber electrode in the NAcs. (**c**) A typical blood oxygen-level-dependent (BOLD) signal time course evoked by DBS and recorded in the NAcs close to the electrode tip. (**d**) Electrical stimulation functional magnetic resonance imaging (Es-fMRI) BOLD maps (*n*=6, *P<*0.001, cluster size 14). Color code denotes the statistical significance (*T*-value). (**e**) Relative activation volumes of all regions showing statistically significant activation. AM, amygdala; CPu, caudate putamen; mPFC, medial prefrontal cortex; Pon, pons; Rx, retrosplenial cortex; Sept, septum; Si, substantia innominata. (**f**) Activation probability maps. Color code denotes the probability of activating a region by NAc stimulation (*n*=6, individual maps thresholded at *P<*0.001, cluster size 14).

Using a biophysical model, we first investigated the efficacy of the DBS paradigm in activating the target region and patterned the electric field potentials within the NAcs target region. This model suggests only local stimulation around the stimulation tip and no widespread stimulation outside the NAcs (Figure 3a). However, such a biophysical model can only simulate local electrical stimulation effects; however, the NAcs is part of a network that is likely affected by DBS.^31^ To investigate the functional consequences of electric microstimulation of the NAcs on the network level, we used es-fMRI. To this end, we applied in the scanner a stimulation protocol comparable to the one used in the behavioral study, with two modifications. First, carbon-fiber electrodes were used to preserve the quality of MR images, allowing functional activations in the vicinity of the implanted electrode to be recorded. Figure 3b shows a representative anatomical T2-weighted image with the location of a carbon-fiber electrode in the NAcs. Second, we stimulated the NAcs in an ON–OFF paradigm during scanning in order to increase the statistical power of the analysis, raising the sensitivity to detect brain regions influenced by DBS. Robust blood oxygen-level-dependent (BOLD) signals in the NAcs were routinely obtained with the above protocol in all tested animals. Figure 3c shows typical BOLD signal time course evoked by DBS and recorded in the NAcs close to the electrode tip. We then computed the voxel-wise probability of brain-wide activation during DBS of the NAcs for all rats. The resulting activation maps in Figure 3d are color-coded and thresholded at *P<*0.01 (corrected) with a cluster size of 14 or more voxels. The observed clusters relate to both shell and core of the NAc, mPFC including the IL and septum. Further, we calculated the relative activation volumes of all regions showing statistically significant activation. This region of interest analysis reflects the average of the activation obtained in each individual animal and, therefore, is less susceptible to voxel-wise interindividual variability. Doing so, we found increased activity in further brain sites including the CPu (Figure 3e). To account for the intra-individual variability in fMRI patterns, we finally computed the voxel-wise probability of activation (Figure 3f) as the proportion of subjects showing a significant activation in a particular location during DBS of the NAc shell.

We then asked whether the activated brain sites in this network are also critical for the observed paradoxical effect of DBS. We performed bilateral chronic-continuous DBS in the CPu and IL cortex in alcohol-dependent rats. However, none of the studied brain sites led to any changes in relapse-like drinking behavior (Supplementary Figure 1), confirming that the NAcs is the critical brain site in the network that mediates the paradoxical effect.

#### The effect of DBS depends on accumbal DA levels

**Figure 4:**
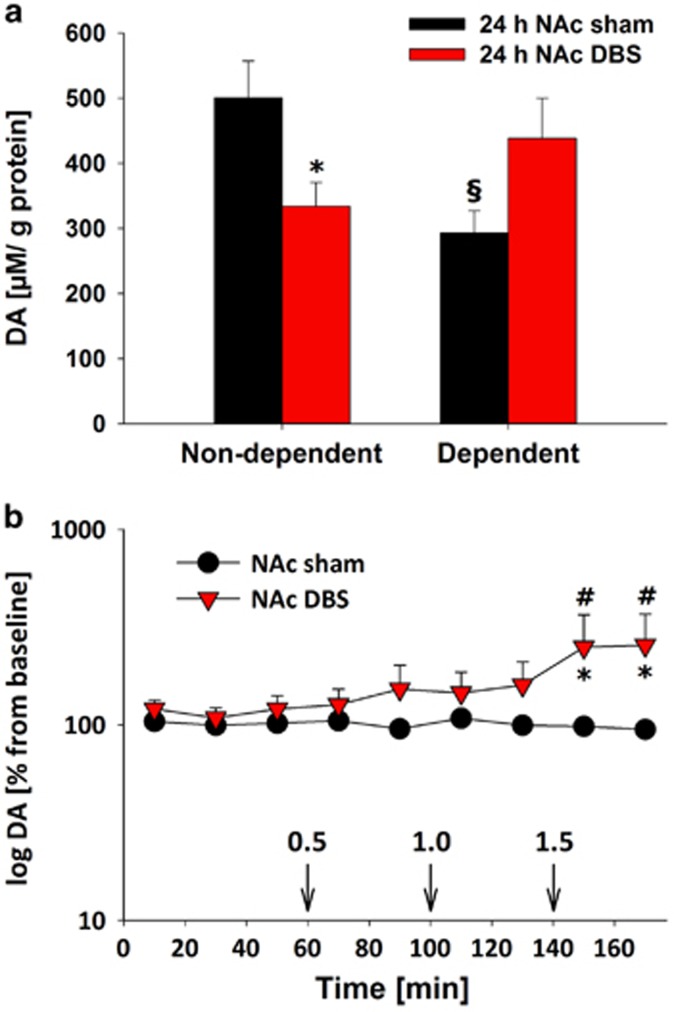
Enhanced dopamine (DA) levels after 24-h DBS and alcohol re-exposure. (**a**) Tissue DA contents in the nucleus accumbens (NAcs) of alcohol-non-dependent and -dependent rats following either 24-h sham stimulation (24 h NAc sham, *n*=8–9) or NAcs DBS (24 h NAc DBS, *n*=6). (**b**) Extracellular DA levels during either the NAc sham stimulation (*n*=7) or bilateral NAc DBS (*n*=6) and following intraperitoneal administration of ascending alcohol doses (0.5, 1 and 1.5 g kg^−1^, arrows) in alcohol-dependent, 3-week abstinent rats. DBS started at the time point 0 and continued throughout the sample collection. Data are presented relative to baseline DA levels (100%), which was defined as the mean of the last four dialysate samples collected before DBS started. * indicates significant difference from the control sham group, § indicates significant difference to respective non-dependent rat group, # indicates significant differences from the baseline condition. Data are presented as means ±s.e.m., *P<*0.05.

Next, a neurochemical analysis was conducted on alcohol-non-dependent and -dependent rats following either sham stimulation or DBS of the NAcs. We studied the main neurotransmitter systems of the NAc and found no differences in tissue glutamate and GABA levels for all tested conditions (Supplementary Table 1). Serotonin was also not affected; however, tissue DA levels were altered. In dependent versus non-dependent rats, we found an opposing regulation of DA by DBS (factor stage × stimulation interaction: F(1,23)=2.7; *P<*0.01). In alcohol-non-dependent rats, NAcs DBS led to a significant reduction in DA (*P<*0.05), whereas, in contrast, NAcs DBS in dependent rats induced an almost significant increase (*P*=0.058) in accumbal DA levels. Of note, accumbal DA levels in dependent rats were significantly smaller than in non-dependent rats (*P<*0.05; Figure 4a).

Using an *in vivo* microdialysis approach, we sought to determine the effects of NAcs stimulation on DAergic neurotransmission at the stimulated site, as well as the influence of alcohol administration on such stimulation, in dependent rats. We first collected samples under baseline conditions and then under NAcs DBS or sham control conditions. This was followed by the application of ascending alcohol doses (0.5, 1 and 1.5 g kg^−1^) along with DBS and sham stimulation. When compared with sham-stimulated alcohol-dependent rats, we found that in NAcs-stimulated animals alcohol led to a significant increase in DA release as compared with baseline and sham stimulation conditions (significant group × time interaction (F(9,81)=2.1, *P<*0.05; Figure 4b).

## Discussion

There is some clinical evidence from case reports of an effect of DBS in alcohol dependence. So far, five patients in total who received bilateral DBS of the NAc showed a reduction in relapse and alcohol consumption.^32^ However, controlled clinical trials with adequate numbers of patients, as has been conducted for DBS in Parkinson's disease,^33^ are still lacking. The lack of clinical trials and our limited knowledge of methodological issues and the neurobiological foundation of DBS in alcohol dependence call for translational experiments to validate these case reports. Here we report on chronic-continuous DBS in an animal model of alcohol dependence in which rats exhibit relapse-like drinking behavior (ADE) after a period of abstinence.^19, 20, 24^ The experimental parameters and setting were designed in a way to best mimic the human situation. As such, (i) we used for, we believe, the first time chronic-continuous DBS—in previous animal work only acute DBS was applied,^11, 12, 13^ (ii) we performed bilateral stimulation of the NAcs ^5, 9, 10^ and other brain regions with the same neurostimulator device (Medtronic Interstim II) and stimulation parameters as used in humans and (iii) we examined the effects of DBS in non-dependent (after the first deprivation) and alcohol-dependent rats (in long-term alcohol-drinking rats that experienced multiple ADEs).^2^

To our surprise, we observed a paradoxical effect of enhanced alcohol consumption during an ADE in alcohol-dependent rats. In non-dependent rats, the occurrence of an ADE was diminished, consistent with a previous report.^12^ In order to provide an explanation for the observed paradoxical effect, we did a network analysis and neurochemical measurements. We were able to confirm that only chronic-continuous electrical stimulation of the NAcs led to a paradoxical effect, whereas DBS in other brain sites such as the IL cortex and CPu did not influence the ADE. One possible explanation for the paradoxical effect stems from our neurochemical studies. We demonstrated, in sham-stimulated alcohol-dependent rats, decreased baseline contents of DA and impaired reactivity of the accumbal DA system to ethanol stimulation, relative to non-dependent controls. DBS within the NAcs led to enhanced overall levels of DA preceding the occurrence of behavioral effects and DBS led to augmented alcohol-induced DA release. These neurochemical findings suggest that chronic-continuous DBS in the NAcs may increase alcohol 'liking' and thereby promote relapse behavior. Although these translational experiments may not perfectly reflect the human situation, the results call for a controlled clinical trial in a sufficient number of heavily dependent and non-treatment-responsive patients. Furthermore, with our study we cannot rule out the possibility that DBS to other NAc subregions such as the NAc core might have yielded different results. Most clinical DBS reports on addiction published so far^5, 9, 10^ indicate that the putative human equivalent to the rodent NAcs was targeted. Still, systematic analyses on the exact target point within the NAc formation that could inform about any site-specific effects are missing.

We used the ADE rat model comprising long-term alcohol consumption with repeated deprivation phases to study the effects of NAc brain stimulation on relapse-like alcohol-drinking behavior. Alcohol-dependent rats that derive from this model show a robust ADE that is characterized by compulsive alcohol drinking, that is, increased incentive salience and hedonic impact of alcohol as demonstrated here and in other studies.^2, 20, 21, 22, 34^ This model has good predictive validity, for example, anti-relapse drugs that reduce relapse behavior in humans also reduce the ADE.^34, 35, 36^ Corticotropin-releasing hormone receptor 1 antagonists that were proposed for relapse prevention but failed in human studies^37^ did not affect the ADE in our model,^38^ showing that negative outcomes can also be predicted by this model. Therefore, the paradoxical effect observed here may also be predictive, at least in some individuals, for the human condition; indeed, a patient who recently received NAc DBS in our clinic showed a worse outcome (Wolfgang Sommer, personal communication).

As the application of DBS in the clinic consists of a chronic-continuous stimulation protocol, we continuously stimulated the NAcs and other brain areas of dependent rats starting 3 days before alcohol re-exposure and continuing during the four post-abstinence days. By means of our drinkometer system,^20, 21^ the drinking pattern and fluid consumption during brain stimulation was measured at high temporal resolution. Fourier analysis revealed that DBS rats exhibited a decreased frequency of drinking approaches, which is interpreted as decreased incentive salience to alcohol ('wanting').^20, 21, 22^ However, those drinking approaches were characterized by an increased alcohol intake (represented by higher amplitude), which in turn reflects increased alcohol 'liking'.^20, 21, 22^ The published case reports^8, 10, 31^ show that dramatic decreases in craving and alcohol 'wanting' is one psychological component of craving.^39^ Obviously, any attempt to extrapolate from animal data to the clinical condition should be cautiously considered; however, the attenuation of incentive salience to alcohol following DBS of the NAcs in the present study might, hence, be in line with the clinical data. If so, future controlled clinical trials should also consider measuring 'wanting' and 'liking' aspects of addictive behavior over extended follow-up periods.

It is suggested that alcohol 'wanting' is generated by a large and distributed brain system, whereas alcohol 'liking' is generated by a smaller set of hedonic hotspots within the limbic circuitry, especially in the NAcs.^40^ Interestingly, our post-mortem neurochemical analysis suggests that before behavioral DBS effects, enhanced DA levels occur within the NAcs following DBS in alcohol-dependent rats—an effect that is even augmented by alcohol, as shown by our microdialysis study. Enhanced DA release in the NAcs might be indicative of and promote enhanced 'liking‘.^40, 41^ These findings provide at least one possible neurobiological explanation for the observed increased 'liking' during DBS. It should be noted that chronic DBS was not tested in non-dependent rats. Hence, it is unclear whether an altered incentive salience and hedonic impact of alcohol is specifically linked to alcohol dependence.

In conclusion, we suggest that DBS in the NAcs can produce incentive salience to alcohol reward via enhanced DA release, and can thereby promote relapse.

## Figures and Tables

**Table 1:** Paradoxical effect of DBS on alcohol relapse-like drinking (ADE) measured by the drinkometer device

*Treatment*	*Fourrier coefficients*	*Water*	*5% EtOH*	*10% EtOH*	*20% EtOH*
*Baseline*					
	Amplitude Frequency	0.500 0.046	0.004 0.044	0.005 0.044	0.005 0.046
					
*ADE, day 1*					
Sham	Amplitude Frequency	0.080 0.044	0.016^a^ 0.046	0.016^a^ 0.616^a^	0.003 0.466^a^
DBS	Amplitude Frequency	0.400^b^ 0.052	0.026^a^ 0.220^a^^b^	0.030^a^ 0.562^a^	0.012^a^^b^ 0.080^b^
					
*ADE, days 2–4*					
Sham	Amplitude Frequency	0.170 0.044	0.020^a^ 0.042	0.009 0.230^a^	0.003 0.184^a^
DBS	Amplitude Frequency	0.210 0.031	0.020^a^ 0.044	0.017^a^^b^ 0.044^b^	0.004 0.087^a^^b^

Abbreviations: ADE, alcohol-deprivation effect; DBS, deep-brain stimulation; NAcs, nucleus accumbens shell.

Data are given for alcohol-dependent rats in the sham-stimulated control group (*n*=8) and in the bilateral NAcs DBS group (*n*=5). Chronic-continuous bilateral NAcs stimulation started 3 days before the end of the abstinence phase and continued for four post-abstinence days. The table shows the differential effects of chronic-continuous DBS on the drinking patterns. The model parameters derived from the Fourier coefficients describe the maximal peak of water/ethanol intake during 5-min intervals (amplitude: in ml kg^−1^ of body weight for water, and in grams of pure alcohol per kilogram of body weight for each ethanol solution) and the number of maximal intake-peak occurrences in 1 h (frequency). The Fourier coefficients calculated for the last days of baseline drinking (Baseline), the first post-abstinence day (ADE, day 1) and the average of the successive three post-abstinence days (ADE, days 2–4) in both control sham-stimulated and DBS rats are displayed. Data are presented as means ±s.e.m., *P<*0.05.

a Indicates significant differences from the baseline condition.

b Indicates significant difference from the control sham group.
