# Supplementary material for: Paradoxical augmented relapse in alcohol-dependent rats during deep-brain stimulation in the nucleus accumbens
Source: Transl Psychiatry. 2016 Jun 21;6(6):e840–. doi: 10.1038/tp.2016.100 (PMC4931598; doi:10.1038/tp.2016.100)
Supplement: Supplementary Information [file tp2016100x1.docx]

**Supplementary Information To**

**Paradoxical augmented relapse in alcohol dependent rats during deep brain stimulation in the nucleus accumbens**

Ravit Hadar, Valentina Vengeliene, Elizabeth Barroeta Hlusicke, Santiago Canals, Hamid R Noori, Franziska Wieske, Julia Rummel, Daniel Harnack, Andreas Heinz, Rainer Spanagel^*^, Christine Winter^*#^

**Supplementary results**

**Supplementary Figure 1.** Effect of DBS in CPu and IL cortex on alcohol relapse-like drinking. Total ethanol intake (g/kg/day) before and after an alcohol deprivation period of 3 weeks in alcohol dependent rats in the sham-stimulated control group (n=6-7) and in either (A) the bilateral CPu (coordinates were AP: +0.5, ML: ±2, DV: -5.4 from bregma) DBS group or (B) the bilateral IL cortex (coordinates were AP: +3, ML: ±0.75, DV: -5 from bregma) DBS group. The last week measurement of ethanol intake is given as baseline drinking – “B”. Chronic-continuous bilateral stimulation started three days before the end of the abstinence phase and continued for four post-abstinence days. Post-abstinence drinking (ADE) is shown as an average alcohol intake during the whole stimulation period. Data are presented as means ± S.E.M.

| **Brain region** | **Transmitter** | **Stimulation** | **Transmitter content (µM/g protein)** | |
| --- | --- | --- | --- | --- |
|  |  |  | **Non -dependent** | **Dependent** |
| **mPFC** | **DA** | sham  DBS | 9.6 ± 1.3  6.6 ± 1.2 | 15.8 ± 2.4 §  15.8 ± 3.1 § |
|  | **5-HT** | sham  DBS | 36.8 ± 3.4  41.2 ± 10.2 | 37.9 ± 2.9  43.5 ± 4.8 |
|  | **Glu** | sham  DBS | 114.5 ± 4.1  112.2 ± 3.3 | 86.6 ± 4.5 §  91.0 ± 6.1 § |
|  | **GABA** | sham  DBS | 25.3 ± 1.4  22.9 ± 1.2 | 13.1 ± 0.5 §  12.3 ± 1.1 § |
| **NAc shell** | **DA** | sham  DBS | 500.7 ± 56.5  333.9 ± 36.8* | 293.1 ± 34.2 §  438.6 ± 61.4*§ |
|  | **5-HT** | sham  DBS | 33.0 ± 8.4  33.6 ± 7.3 | 20.7 ± 4.5  45.1 ± 4.8 |
|  | **Glu** | sham  DBS | 86.6 ± 4.6  91.0 ± 2.4 | 54.5 ± 2.5 §  68.6 ± 8.9 § |
|  | **GABA** | sham  DBS | 42.4 ± 3.8  41.8 ± 2.2 | 13.2 ± 0.9 §  17.0 ± 2.0 § |
| **CPu** | **DA** | sham  DBS | 690.5 ± 66.7  650.5 ± 63.3 | 506.5 ± 47.5 §  500.1 ± 27.4 § |
|  | **5-HT** | sham  DBS | 20.8 ± 2.8  23.7 ± 5.2 | 22.6 ± 4.3  34.0 ± 2.3 |
|  | **Glu** | sham  DBS | 99.8 ± 2.8  96.5 ± 3.3 | 71.4 ± 6.8 §  70.3 ± 7.4 § |
|  | **GABA** | sham  DBS | 29.3 ± 1.9  26.4 ± 1.5 | 12.0 ± 1.3 §  15.7 ± 3.3 § |

**Supplementary Table 1.** **The effect of DBS depends on accumbal dopamine levels.** Tissue content of dopamine (DA), 5-hydroxytryptamine (5-HT), glutamate (Glu) and γ-Aminobutyric acid (GABA) in the mPFC, NAcs and CPu of alcohol non-dependent and dependent rats following 24 h sham or DBS stimulation. * indicates significant difference from the control sham group, § indicates significant difference from the respective non-dependent group.
